# Omics-guided therapeutic translation in acute traumatic brain injury

**DOI:** 10.3389/fphar.2026.1968318

**Published:** 2026-10-28

**Authors:** Logan R. Van Nynatten, Enis Cela, Aleah Wilson, Ian Ball, Douglas D. Fraser

**Affiliations:** 1 Division of Critical Care Medicine, Department of Medicine, Schulich School of Medicine and Dentistry, Western University, London, ON, Canada; 2 Physiology and Pharmacology, Western University, London, ON, Canada; 3 Epidemiology and Biostatistics, Western University, London, ON, Canada; 4 London Health Sciences Centre Research Institute, Western University, London, ON, Canada; 5 Division of Critical Care Medicine, Department of Pediatrics, Schulich School of Medicine and Dentistry, Western University, London, ON, Canada; 6 Clinical Neurological Sciences, Western University, London, ON, Canada; 7 Children’s Health Research Institute, London, ON, Canada; 8 GSK Chair in Clinical Pharmacology, Western University, London, ON, Canada

**Keywords:** drug repurposing, endotypes, neuroinflammation, omics, precision medicine, systems biology, traumatic brain injury

## Abstract

Decades of trials evaluating secondary-injury targeted therapeutics in traumatic brain injury (TBI) have failed almost without exception, typically concluding that the intervention was ineffective. We argue that these failures stem largely from methodological limitations and should be reconceptualized. TBI pathobiology has traditionally been studied, stratified, and treated using single markers, isolated mechanisms, and discrete timepoints, despite being heterogeneous, compartmentalized, and temporally dynamic. High-dimensional omics, interpreted at the level of biological pathways rather than individual analytes, offers a direct means of addressing this complexity. Focusing on the acute phase of moderate-to-severe TBI, we define four recurrent failure modes in TBI therapeutic translation: the wrong patient, the wrong sample, the wrong window, and the wrong mechanism. For each, we identify targeted omics and pathway-level strategies that can mitigate these limitations and summarize what such approaches have revealed about TBI pathobiology. Finally, we argue that pathway-based predictive enrichment, combined with drug repurposing, is a promising near-term strategy that may improve the odds of detecting a real treatment effect where unselected trials have not.

## Introduction

Traumatic brain injury (TBI) is among the most common and costly neurological conditions worldwide, remaining a leading cause of death and long-term disability, particularly among those under the age of 45 (James et al., 2019; Dewan et al., 2016; Dewan et al., 2018). Its pathophysiology is conventionally divided into two phases. The primary injury (the mechanical disruption of tissue at the moment of impact) is, by definition, complete before the patient reaches care and is not therapeutically modifiable (Haddad and Arabi, 2012). The clinically actionable disease is the secondary injury that unfolds over the subsequent hours, days, and weeks. This is a self-amplifying cascade of excitotoxicity, mitochondrial and metabolic failure, oxidative stress, blood-brain barrier (BBB) breakdown, and a sustained neuroinflammatory response orchestrated by activated microglia and astrocytes, which is amplified by infiltrating peripheral leukocytes (Simon et al., 2017; Jassam et al., 2017; Najem et al., 2018). Many other processes add to this damage, including ischemia, cerebral edema, cortical spreading depolarization, coagulopathy, and systemic insults such as hypoxia, hypotension, and fever, so the number of factors that shape final outcome is very large (Maas et al., 2017; Cela et al., 2026). Given that these processes evolve within a treatable time window and are individually druggable in experimental models, TBI is regarded as a tractable target for pharmacological intervention. This framing has driven a conceptual shift from viewing TBI as an isolated traumatic event to understanding it as a chronic disease process with ongoing, modifiable biology (Masel and DeWitt, 2010).

Despite extensive pathobiology and clinical trials research, no effective targeted biologic therapy exists for TBI (Guo et al., 2024). Even surgical management offers minimal success. Decompressive craniectomy lowers mortality in refractory intracranial hypertension but shifts survivors toward severe disability and vegetative states (Hutchinson et al., 2016). Earlier craniectomy has been associated with worsening functional outcomes despite controlling intracranial pressure (Cooper et al., 2011). Pharmacological neuroprotection has failed across multiple mechanistic classes. Corticosteroids increased mortality in one of the largest TBI trials ever conducted (CRASH Trial Collaborators, 2004). Progesterone, a pleiotropic neurosteroid with anti-inflammatory and anti-edema properties, showed no benefit in two independent trials (Skolnick et al., 2014; Wright et al., 2014). Erythropoietin (Nichol et al., 2015), citicoline (Zafonte et al., 2012), the cannabinoid antioxidant dexanabinol (Maas et al., 2006), and hypothermia (Cooper et al., 2018; Andrews et al., 2015) each yielded neutral (or harmful) results in separate studies. The few trials with weakly positive signals do so in narrow, well-defined populations, such as amantadine for accelerating the pace of functional recovery in post-traumatic disorders of consciousness (Giacino et al., 2012), and tranexamic acid for reducing head-injury death in a prespecified subgroup with mild-to-moderate injury (Crash-3 Trial Collaborators, 2019). Yet, their clinical use has outpaced the supporting evidence.

These treatment shortcomings are routinely attributed to the agents or to deficiencies in trial design. These criticisms have merit, but a more fundamental pattern recurs. With few exceptions, each trial tested a single mechanism, in a population defined by clinical severity alone, often at a single dose delivered within a single time window, against a syndrome that is biologically heterogeneous, anatomically and compartmentally complex, and temporally dynamic (Åkerlund and Ercole, 2023). Several of these trial design problems, including dilution of benefit across mixed populations, the many mechanisms of brain damage, and the importance of the therapeutic window, have also been discussed recently (Imberti, 2025). The field has, in effect, repeatedly asked whether a drug works ‘on average’ in TBI, when the biologically answerable question is which process is active, in which patient, in which neuronal tissue and at which time. Framed this way, the negative trials may indicate not that the drugs are ineffective, but that overly broad patient grouping obscured treatment-responsive subgroups.

High-dimensional omics and pathway-level analyses offer a promising solution to this barrier (Van Nynatten et al., 2026a). High-dimensional omic profiling measures hundreds to thousands of analytes (such as proteins, metabolites, or gene transcripts) simultaneously, and, when interpreted at the level of dysregulated cellular signaling networks, rather than individual molecules, can resolve the heterogeneity, compartmentalization, and temporal structure that single markers cannot (Cela et al., 2026; Van Nynatten et al., 2026b; Van Nynatten et al., 2026d). This review focuses on the acute phase of moderate-to-severe TBI, meaning the first days to weeks after injury when patients are in hospital and most candidate therapies would be given. We use neuroinflammation as a worked example because it is among the most studied and most druggable secondary injury processes, not because it is the only one. In this review we offer the field a framework of recurrent failure modes in TBI therapeutic translation, and pair each with the omics and pathway-level approach that addresses it. We then highlight what omics and informatics have revealed about TBI pathobiology and argue that pathway-based biology merged with drug repurposing offers a promising near-term route to better designed trials, while recognizing that this remains a hypothesis to be tested prospectively.

## The wrong patients: biological heterogeneity

TBI is a clinical syndrome, not a molecular diagnosis. TBI encompasses injuries of fundamentally different biomechanics (blunt, penetrating, and blast injury) that produce distinct and often co-existing pathoanatomical lesions (Saatman et al., 2008). Diffuse axonal injury, focal contusion, and hemorrhage may each have their own cellular response. Superimposed on this are host factors such as age, sex, genetic background, and comorbidity that shape inflammatory and repair responses (Xiong et al., 2019). These host factors matter for enrichment as well. Older age, for example, is linked to the early-inflammatory immune endotype described below (Samanta et al., 2024), and sex, genetic variants such as APOE ε4, pre-injury medications, and in-hospital treatments such as sedation, temperature control, transfusion, and surgery may all alter both pathway activity and the response to a drug. Any molecular enrichment strategy will need to account for these factors, either by including them in selection models or by testing them as effect modifiers. The instruments used to classify TBI, including the Glasgow Coma Scale (GCS), pupillary reactivity, or CT imaging, reproducibly quantify the magnitude and gross anatomy of injury, thereby informing validated prognostic models (Steyerberg et al., 2008; MRC Crash Trial Collaborators, 2008). Yet, they are insufficient to identify the underlying pathobiology. Two patients indistinguishable in clinical presentation can mount divergent molecular responses and, plausibly, require different treatments. The heterogeneity of TBI is itself widely regarded as the central barrier to effective therapy (Maas et al., 2017; Masel and DeWitt, 2010; Saatman et al., 2008). Stratifying by clinical severity therefore groups together patients who share a prognosis but not a mechanism, which is precisely the wrong basis on which to allocate a mechanism-specific drug.

Single-analyte markers, despite clinical utility, do not resolve this problem. Glial fibrillary acidic protein (GFAP; astrocytes) and ubiquitin carboxy-terminal hydrolase L1 (UCH-L1; neurons) are now available as point-of-care tests to triage the need for CT after mild head injury (Bazarian et al., 2018), and prognostic markers such as neurofilament light chain (NfL; neurons) and S100 calcium-binding protein B (S100B; astrocytes) reliably quantify the severity of structural damage, and carry genuine prognostic information for death and unfavourable functional outcomes (Korley et al., 2022; Helmrich et al., 2022). Yet, the concentration of a structural-injury protein in the blood reports how much tissue was damaged, not which specific inflammatory, vascular, or metabolic programs are currently active and therefore which therapeutic might be beneficial. A marker of injury magnitude is not a marker of injury mechanism. Worse, stratifying a multi-pathway syndrome one protein at a time is underpowered by design (Subramanian et al., 2005). Any single analyte captures only a sliver of the relevant biology, so associations found this way often fail to replicate. This problem has long plagued the candidate-biomarker literature in TBI drug development.

### Resolving heterogeneity

High-dimensional profiling is the simultaneous measurement of thousands of molecular markers (proteins, transcripts, lipids, or metabolites) in a single sample, producing a broad, system-wide view of biology (Emmert-Streib, 2022). Its power lies less in the length of that list than in what the markers reveal together. Because each measured marker can be mapped to the pathways in which it acts, the coordinated rise or fall of many members of the same pathway exposes which biological processes are actively shaping a patient’s pathology (Figure 1). Summarized as pathway-level signatures (through enrichment analysis against curated databases, or data-driven network modules), these measurements are not only possibly more reproducible across cohorts than any single analyte, but also more actionable, because a dysregulated signaling pathway is itself a potential candidate drug target (Subramanian et al., 2005; Chuang et al., 2007; Lee et al., 2008). This motivates a new possible basis for endotyping: grouping patients by the pathways actively driving their disease rather than by clinical severity or by clusters of individual proteins, so that an undifferentiated “TBI” resolves into a small number of mechanism-defined molecular endotypes. An endotype refers to a subgroup defined by a distinct underlying biological mechanism, whereas subphenotype refers to a subgroup identified from clinical or molecular data before its mechanism is confirmed (Gordon et al., 2024). The pathway, not the marker, becomes the stable unit of heterogeneity (Van Nynatten et al., 2026d). These endotypes also set up better interventional trials. Instead of enrolling everyone, a trial can include only the patients whose biology the drug actually targets (Van Nynatten et al., 2026a). Integrating this profiling with clinical, imaging, and traditional diagnostic data is a further advance, aligning molecular pathophysiology with each patient’s characteristics in support of a precision-medicine approach (Daley et al., 2022; Li et al., 2025). Large-scale initiatives such as TRACK-TBI and CENTER-TBI are already applying this thinking, working to modernize the diagnosis, classification, and treatment of TBI beyond traditional injury-severity labels (Maas et al., 2017).

**FIGURE 1:**
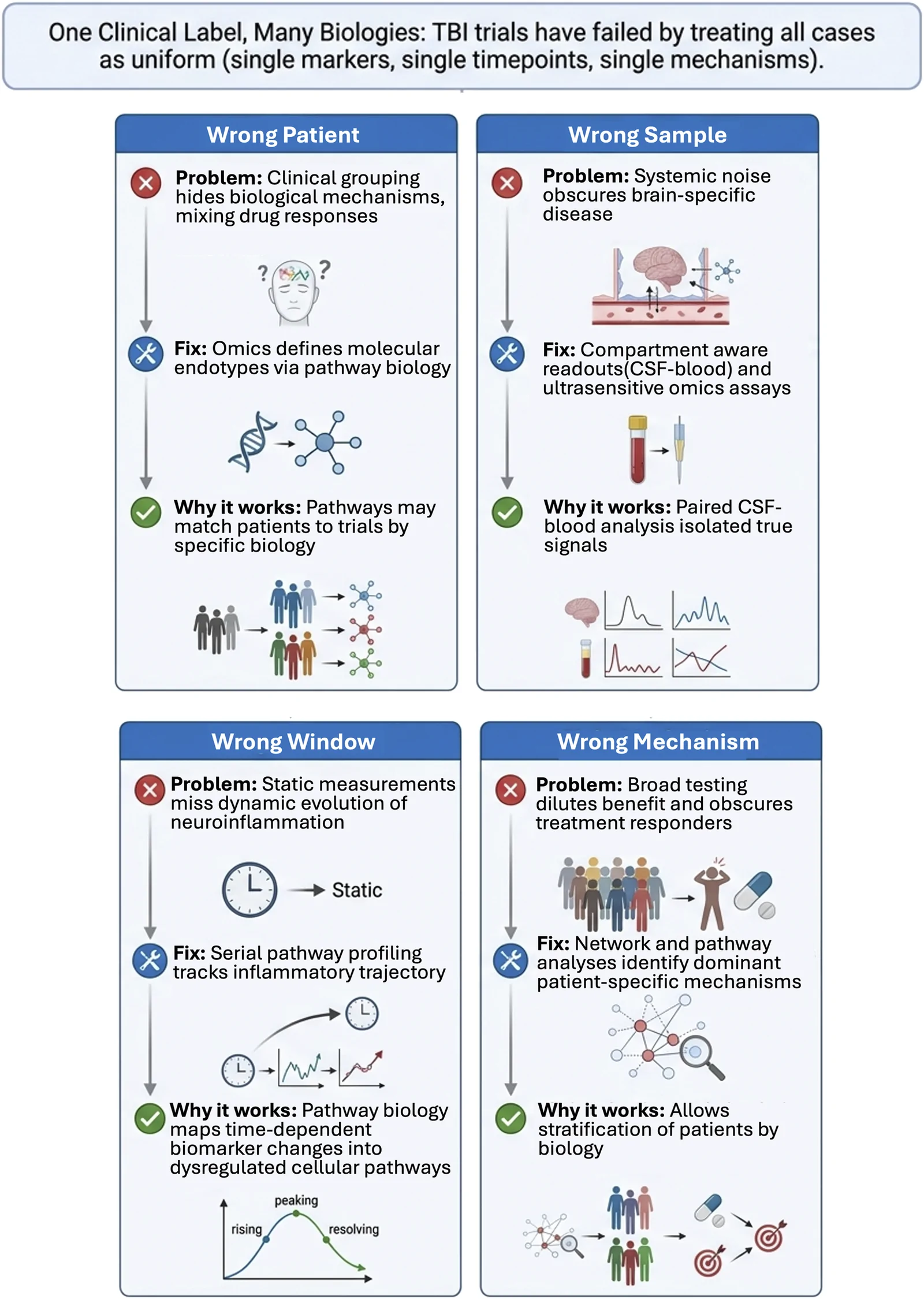
Failure Modes In Therapeutic Translation In Traumatic Brain Injury. High-dimensional omics and pathway biology address the four recurrent failure modes of traumatic brain injury (TBI) trials. Because TBI is one clinical label spanning many distinct biologies, trials have repeatedly failed by treating it as uniform, using single markers, single timepoints, and single mechanisms. Each column pairs a failure mode with its omics or pathway-biology fix. Each column moves from the failure mode described in the text (the wrong patient, sample, window, or mechanism) to its corrected form (the right patient, sample, window, or mechanism). Wrong patient: grouping by clinical severity (GCS, CT) shares prognosis but not mechanism, so mechanism-specific drugs reach a biologically mixed crowd; omics instead resolves patients into molecular endotypes defined by their active pathways. Wrong sample: the disease sits behind the blood-brain barrier, so a blood measurement conflates brain-derived, systemic, and barrier-state signals; compartment-aware, pathway-level readouts (CSF, microdialysis, ultrasensitive blood assays) separate CNS signal from systemic noise. Wrong window: neuroinflammation evolves from an early neurotoxic phase to later repair, so a single snapshot cannot distinguish a rising response from a resolving one; serial pathway profiling captures the trajectory over time. Wrong mechanism: a single drug tested in all-comers dilutes real benefit and may harm non-responders (i.e., corticosteroids); network and pathway analysis identifies the dominant active mechanism in each patient and its matched target (drug-repurposing). Across all four, resolving the active pathway rather than any single analyte is what enables pathway-matched, predictive-enrichment trials. (GCS, Glasgow Coma Scale; CT, computed tomography; CSF, cerebrospinal fluid; CNS, central nervous system).

## The wrong sample: the compartment problem

A distinctive obstacle in developing therapeutics that target neuroinflammation in TBI is a consequence of the syndrome occurring behind the blood-brain barrier, whereas most studies utilizing omics sample only peripheral blood (Lindblad et al., 2021). The BBB is a selective interface that, under physiological conditions, isolates the central nervous system (CNS) from the circulation. TBI disrupts it, often in a biphasic and regionally heterogeneous pattern, opening the barrier to bidirectional exchange of proteins, immune cells, and signaling molecules (Chodobski et al., 2011). Early disruption follows mechanical shearing of the microvasculature and is then sustained over hours to days by inflammatory cytokines, matrix metalloproteinases, and complement activity that break down tight junctions. The barrier gradually reseals as tight junctions and pericyte coverage recover, although leak can persist for weeks to years in some patients (Shlosberg et al., 2010). In practical terms, the relationship between an intracranial process and its plasma readout is mediated by a barrier whose permeability changes over the very period during which plasma markers are quantified. Hence, the same traumatic injury can produce different blood signals at different times, and a rising biomarker concentration may reflect either increasing brain production or increasing barrier leak.

A circulating protein measured after TBI can arise from at least three distinct sources, which single-marker assays cannot distinguish. The protein may: 1) originate in the injured brain and reach blood through BBB permeability or the circulation of cerebrospinal fluid through the meninges and into systemic circulation; 2) be produced by the systemic immune and stromal response to the injury, which is intense in the polytrauma patients who dominate severe-TBI cohorts; or 3) be a constitutive plasma protein unrelated to the CNS. Clinically deployed markers illustrate this hazard. Although GFAP and UCH-L1 are brain-enriched, both are detectable after extracranial trauma, and GFAP in particular can be elevated by orthopedic and other systemic injury, limiting its specificity for neuronal injury (Helmrich et al., 2022; Lindblad et al., 2021). Blood concentration, in isolation, cannot separate brain origin, systemic origin, and barrier state.

Methods that sample the CNS compartment directly expose what blood obscures. Cerebrospinal fluid (CSF) proteomics, and intracerebral microdialysis, which recovers small molecules, cytokines, and other macromolecules from the extracellular space of the injured brain, provide readouts of intracranial metabolic processes and neuroinflammation that plasma may not detect (Zeiler et al., 2017). Paired studies comparing CSF and serum proteomes after severe TBI show that some inflammatory signals appear in both compartments while others are confined to one, and that markers of blood-brain barrier breakdown rise in proportion to its permeability (Lindblad et al., 2021). Intracranial readouts represent any measure taken from within the cranial compartment. This includes imaging (e.g., translocator-protein PET, diffusion tensor imaging), physiological monitoring (e.g., intracranial pressure, brain tissue oxygen), and molecular measures from CSF or microdialysate. The value of an intracranial readout is well illustrated by the phase II trial of the interleukin-1 (IL-1) receptor antagonist anakinra in severe TBI. There, microdialysis demonstrated drug-target engagement within the CNS and a shift in the cerebral cytokine and chemokine milieu that a peripheral venous measurement alone could not have established (Helmy et al., 2014). Obtaining these samples is itself a major barrier. CSF is usually available only when an external ventricular drain is placed for clinical reasons, microdialysis is limited to a few specialized centres, and consent in the early window usually depends on substitute decision makers. The patients and timepoints that matter most are therefore often the hardest to sample. Confirming that a drug reaches and modulates its target within the CNS, rather than merely altering a blood marker, is a problem unique to neurotherapeutics, and one that omics applied to the right compartment is positioned to solve.

### Resolving tissue compartments

Partial mitigation is now emerging from the analytical side (Figure 1). The historical obstacle to reading intracranial biology from blood has been sensitivity. Brain-derived proteins enter the circulation at very low concentrations, particularly when the BBB is relatively intact, and conventional proteomics tools (i.e., enzyme-linked immunosorbent assay (ELISA) or mass spectrometry (MS)) do not reliably detect them against the high-abundance plasma background (Chen et al., 2021). Two advances narrow this gap. First, ultrasensitive multiplex platforms (most notably nucleic acid-linked immunosandwich assays (NULISA), which reach attomolar detection) can now quantify low-abundance, brain-enriched proteins in plasma, and dedicated CNS panels are explicitly designed to capture brain-derived signals (Feng et al., 2023). For example, NULISA CNS panels quantify low-abundance, brain-enriched analytes that conventional assays struggle to detect in blood, including phosphorylated tau species (p-tau181, p-tau217, p-tau231), α-synuclein and its Ser129-phosphorylated form, and TDP-43, directly in plasma (Gong et al., 2025). The discriminating power is striking: on this platform, plasma p-tau217 tracks brain amyloid status with an area under the curve of 0.96 and roughly 94% concordance with amyloid PET (Gong et al., 2025), showing that a peripheral assay can now read a pathology-specific, CNS-restricted signal with near-imaging fidelity. Crucially, this is a gain in certainty as much as in sensitivity. Proteins that were previously undetectable, or indistinguishable from the high-abundance plasma background, can now be measured reliably, thus a brain-enriched signal in blood can be interpreted with far greater confidence that it is genuinely neural in origin rather than noise. Second, breadth has expanded in parallel. Proximity extension assay (PEA) panels now measure more than 5400 proteins per sample, and proteome-scale plasma profiling has proven able to resolve pathway-level perturbations (across cytokine, complement, and other networks) rather than isolated analytes (Sun et al., 2023; Wik et al., 2021; Assarsson et al., 2014). Together, depth and breadth make it increasingly feasible to interrogate for dysregulated neuroinflammatory and other CNS-relevant pathways from a peripheral blood sample. This does not abolish the compartment problem. A brain-derived protein detected in plasma is still diluted, still subject to systemic and BBB-state confounding, and still an indirect readout. However, it partially relaxes the constraint that intracranial biology can be seen only by sampling CSF, and it reinforces the case for compartment-aware, pathway-level interpretation rather than reliance on CSF access alone.

A legitimate and central question is whether a plasma proteome can report intracranial biology at all, given that the severe-TBI population is dominated by polytrauma patients in whom a large systemic injury response runs concurrently. Two lines of evidence indicate that this confounding, while real, is a design constraint rather than a disqualifying flaw. First, blood and CSF proteomes are partially concordant. In paired severe-TBI samples, a subset of inflammatory and complement signals appear in both compartments and scale with blood-brain barrier permeability, establishing that at least part of the plasma signal is genuinely neural in origin (Lindblad et al., 2021). This concordance is expected to be incomplete, because the brain-to-blood relationship is gated by a barrier of varying permeability, and plasma protein levels reflect CNS regulation only partially even at baseline (Yang et al., 2021). Second, and most directly relevant to peripheral-trauma interference, comparative cohorts that include extracranial-trauma controls can separate a brain-specific signature from the systemic trauma response. Discovery plasma proteomics distinguishes isolated TBI, polytrauma with TBI, and polytrauma without TBI (Cralley et al., 2023), differentiates brain from torso injury (Tachino et al., 2024), and in the BIO-AX-TBI cohort resolves proteins that are TBI-specific (e.g., IL-16, CCL2) from those that rise in both TBI and non-TBI trauma (e.g., IL-10, CHI3L1), so that the trauma-general response can be explicitly identified rather than mistaken for CNS biology (Li et al., 2025). Brain-enriched analytes further strengthen this attribution: β-synuclein, expressed almost exclusively in neurons, is scarcely released by extracranial injury and discriminates TBI even within polytrauma cohorts (Halbgebauer et al., 2022), and GFAP retains diagnostic performance for intracranial injury in trauma patients with extracranial lesions, outperforming the less specific S100B (Papa et al., 2014); nonetheless, GFAP and UCH-L1 do carry a measurable extracranial contribution and must be interpreted with that caveat (Helmrich et al., 2022). None of this eliminates the compartment problem. Plasma biomarkers remain diluted, influenced by the blood-brain barrier, and affected by signals from the rest of the body. However, rather than being an unresolvable limitation, it highlights practical steps for future studies: interpret blood biomarkers with these limitations in mind, include extracranial trauma control groups, use high-sensitivity, brain-enriched omics platforms for peripheral blood rather than conventional single-analyte immunoassays, prioritize paired blood-CSF sampling where feasible, and, when evaluating a therapy, confirm CNS target engagement directly (e.g., using microdialysis) rather than relying only on changes in peripheral blood biomarkers.

## The wrong window: temporal dynamics

Neuroinflammation after TBI is not a fixed state but an evolving program, and treating it as static is a recurrent source of translational failure. Microglia and astrocytes do not occupy a single “activated” state. They transition through a spectrum of context-dependent phenotypes whose functional roles change over time. The once-standard dichotomy of pro-inflammatory “M1” and reparative “M2” microglia is now recognized as an oversimplification of a far more heterogeneous and dynamic continuum (Loane and Faden, 2010; Loane and Kumar, 2016; Simon et al., 2017). Broadly, an early response that is predominantly pro-inflammatory and neurotoxic gives way, variably and incompletely, to reparative activity over days to weeks. Critically, this response does not simply terminate. Using translocator-protein PET, microglial activation has been demonstrated in subcortical structures up to 17 years after a single moderate-to-severe injury, in regions remote from the focal damage and is associated with cognitive impairment (Ramlackhansingh et al., 2011). TBI is, in this sense, a disease with a years-long inflammatory tail rather than a self-limited insult (Masel and DeWitt, 2010).

This temporal structure has direct therapeutic consequences that unselected, single-timepoint trials are poorly designed to incorporate. An intervention that is beneficial during the early neurotoxic phase may be inert or harmful during the reparative phase. If mistimed, broad suppression of inflammation risks blocking the resolution and repair signaling on which recovery depends, a plausible contributor to the harm seen with indiscriminate corticosteroids. Yet, the dominant sampling paradigm remains a single measurement, often at hospital admission, from which a static stratification is derived. Proteomic trajectories are therefore likely biased toward structurally stable proteins with slower turnover kinetics, such as neurofilaments and GFAP, that accumulate gradually and persist in circulation over several days (Thelin et al., 2017). In contrast, more transient inflammatory mediators with rapid turnover may be underrepresented under current sampling schedules (Dyhrfort et al., 2019). Earlier and more frequent sampling will therefore be necessary to fully capture the acute inflammatory proteomic landscape. Time of day adds a further layer. TBI disrupts sleep and circadian rhythms, and many inflammatory mediators, cortisol, and circulating immune cells follow a daily cycle that can itself be altered by injury, sedation, and ICU light and noise (Zielinski and Gibbons, 2022). Serial sampling protocols should therefore record, and ideally standardize, the clock time of each sample. An identical analyte concentration can carry opposite biological meaning at day one versus day five. A single snapshot therefore cannot distinguish a patient whose inflammatory response is escalating from one in whom it is resolving, even though these patients may warrant opposite treatments.

### Resolving temporal dynamics

The omics solution is to read the injury longitudinally, treating the biological trajectory rather than any single value as the signal (Figure 1). This is already feasible in human TBI. Serial CSF proteomics reveals a shift from a neutrophil-driven to a reparative program over the first 10 days after injury (Shultz et al., 2022), and serum metabolomics captures a move from glycolytic to excitotoxic amino-acid signatures between days one and four (Banoei et al., 2025), each visible only because the same pathways were tracked over time. Pathways help clinicians to understand the injury trajectory in two complementary ways. First, they are more robust to *when* a sample happens to be taken. Individual proteins rise and fall on their own kinetics, so any single analyte caught at one moment is a poor readout of where the injury is, whereas a whole pathway moves more stably than any of its members (Chuang et al., 2007; Lee et al., 2008). Second, when tracked serially, a pathway separates a patient’s baseline from the direction and speed of change in their biology, quantities that can diverge; a single marker may plateau at a ceiling while the underlying process is still accelerating, whereas the same pathway followed over time shows whether it is rising, peaking, or resolving. Reading the trajectory rather than a value at an arbitrary moment is what lets an intervention be matched to the phase in which its target is active and lets pathway-defined trials use the time-windowed enrollment they require (Van Nynatten et al., 2026b).

## The wrong mechanism: undifferentiated targeting

Even a potent, CNS-penetrant drug fails if it is administered to patients in whom its target pathway is not active. A clinically defined population comprises a mixture of distinct underlying biologies and mechanisms of action, resulting in an average treatment effect that is diluted across this heterogeneous population. A genuine benefit confined to a responsive subgroup may be masked by absence of effect, or by harm, in the remainder. This is ‘*heterogeneity of treatment effect’*, in which the average effect across a population misrepresents, or even inverts, the effect within its subgroups. Averaging over a biologically mixed cohort is precisely the design under which a real, mechanism-specific benefit is least likely to be detected, and it describes the great majority of failed TBI trials (Van Nynatten et al., 2026a). Broad immunosuppression is a cautionary archetype. Corticosteroids suppress inflammation indiscriminately, and in TBI they not only failed but increased mortality (CRASH Trial Collaborators, 2004). This undesirable result is consistent with the dual, phase- and context-dependent role of post-injury inflammation, in which the same pathways that drive secondary injury early also serve clearance and repair later. An agent blind to which of these roles predominates in a given patient at a given time cannot be expected to help on average. The lesson is not that inflammation is the wrong target, but that targeting it without resolution of who, when, and which specific inflammatory pathways are driving neuroinflammation is a therapeutic strategy designed to fail.

### Resolving therapeutic targeting

An illustrative example of this treatment conundrum is the phase II trial evaluating anakinra, an interleukin-1 (IL-1) receptor antagonist, in patients with severe TBI. Rather than suppressing inflammation non-specifically, it targeted a single, early-emerging cytokine pathway (IL-1) whose engagement could be confirmed within the brain compartment (Helmy et al., 2011; Helmy et al., 2014). Whether or not IL-1 antagonism ultimately improves outcomes, the design exemplifies the shift from blunt to targeted intervention. Single-marker selection cannot reliably support this shift, because one analyte rarely identifies which of several co-active pathways predominates in disease progression in an individual patient. Pathway-resolved profiling can overcome this predicament. It identifies the dominant active mechanism and reframes the question from “does this drug work in TBI?” to “does this drug work in the patients whose biology it targets?”.

High-dimensional omics and systems biology are well suited to address undifferentiated targeting. Where a single analyte reports one correlate of injury, integrated proteomic, transcriptomic, and metabolomic profiling captures the many processes active in a patient at any one time. Network- and pathway-level methods then collapse that breadth into the co-regulated modules that drive the disease state. This matters for target selection in two ways. First, looking at how signals move across a connected network, rather than ranking markers one by one, is less prone to the false positives and failures to replicate that plague single-marker studies, and it points to mechanisms rather than mere associations (Jiang et al., 2025). Second, the same network used to identify a dysregulated pathway can also be queried against drug databases to identify compounds that target that pathway, enabling a single analysis to both stratify patients and identify a mechanism-matched therapy (Jiang et al., 2025). In TBI specifically, a systems-biology framing was proposed over a decade ago as the strategy to address the failed single-drug-to-single-target paradigm, explicitly centered on disease-relevant converging pathways as intervention points paired with downstream biomarkers to track target engagement (Zhang et al., 2010). More recent work has begun to deliver on that proposal. Integrated multi-omic analyses of human TBI now resolve the inflammatory, metabolic, and vascular programs underlying injury and recovery and connect them to candidate therapeutic pathways (Wu et al., 2021), and network-proximity methods have been used to nominate repurposed drugs for neurodegenerative disease which may be applicable to TBI from the human interactome (Bykova et al., 2026). These efforts remain early and largely unvalidated in prospective trials, but they establish a core claim. The biological mechanism driving an individual patient’s disease is reflected in the coordinated dysregulation of molecular pathways rather than any single molecule, necessitating network-level analysis instead of single-biomarker measurements.

## Evidence from omics: subphenotypes and convergent pathways

Preliminary omics studies of adult TBI now offer a molecular view of emerging subphenotypes. The first is immune-response subphenotyping. Applying a multiplex inflammatory panel to the CENTER-TBI cohort, with validation in the TRACK-TBI pilot study, investigators derived two immune endotypes that replicated across both cohorts: an early-inflammatory cluster with higher IL-6, IL-15, and monocyte chemoattractant protein-1 (MCP-1), and a pauci-inflammatory cluster (Samanta et al., 2024). The early-inflammatory endotype occurred in older patients and was associated with unfavourable 6-month functional outcome. Importantly for clinical deployment, it could be captured parsimoniously by a combined IL-15 and MCP-1 signature (Samanta et al., 2024). Because previous untargeted attempts at immunomodulation in TBI have not improved outcomes, these subphenotypes may serve as a basis for predictive enrichment in future immunomodulatory trials (Samanta et al., 2024). The use of an independent validation cohort, and the reduction to a measurable two-marker signature, make this among the more credible molecular stratifications available in the field.

The second major subphenotype is derived from multi-omic profiling in severely injured populations that include TBI. Integrating proteomics, metabolomics, and lipidomics resolved two reproducible response endotypes. These aligned with clinical trajectory and, critically, demonstrated heterogeneity of treatment effect (Wu et al., 2021). Prehospital administration of thawed plasma reduced mortality only in endotype-2 patients with TBI, with UCH-L1 emerging as a single-marker proxy for the responsive endotype (Wu et al., 2021). This evidence is proof-of-principle for the central claim that TBI harbors molecular subgroups that modify the effect of a treatment, and that those subgroups can in principle be identified at the bedside.

Other adult cohorts extend this picture while displaying its limits. Unsupervised probabilistic clustering of early clinical, physiological, laboratory, and imaging data from 1728 intensive-care patients in CENTER-TBI resolved six stable subphenotypes distinguished less by presenting injury than by a profile of systemic metabolic derangement (Åkerlund et al., 2022). A subgroup with clinically moderate injury but deranged metabolism fared worse than one with severe presenting neurology and normal metabolism, and adding endotype labels improved the prognostic precision of the IMPACT model (a standard, validated TBI outcome-prediction model based on admission characteristics) (Åkerlund et al., 2022). A subsequent analysis from the same consortium showed that these subgroups are not static but instead follow distinct trajectories over the intensive care stay, reinforcing the point that subgroup assignment depends on when sampling occurs (Åkerlund et al., 2024). Moving from clinical to molecular features, a high-dimensional plasma proteomic study of the BIO-AX-TBI cohort profiled moderate-to-severe TBI on parallel multiplex platforms and identified TBI-specific changes in proteins of neurodegenerative, inflammatory, and cellular-process pathways. Exploratory clustering separated patients into subgroups that mapped onto distinct injury patterns, but not onto age or 6-month functional outcome (Li et al., 2025). Together, these efforts strengthen the molecular case for biologic heterogeneity in TBI while illustrating its current ceiling. Each scheme was derived within a single cohort, the subgroups are defined by different data types and sampling windows, and only some are linked to outcome, let alone to a modifiable treatment effect.

These findings should nonetheless be read with appropriate caution, and the contrast with more mature fields is instructive. In sepsis and the acute respiratory distress syndrome (ARDS), molecular and clinical subphenotyping has been replicated across many cohorts, yet the stability of subgroups across platforms, panels, and timepoints remains an active concern (Calfee et al., 2018; Calfee et al., 2014; Davenport et al., 2016). In TBI the evidence base is thinner still. Few endotype schemes have been replicated in fully independent cohorts, treatment-effect interactions have generally been observed in single cohorts, and subgroup structure is sensitive to the analyte panel and the timing of sampling. Honesty about this gap is essential to the credibility of the enterprise. Table 1 summarizes the principal adult schemes and their current reproducibility status, deliberately distinguishing what has been validated from what remains exploratory.

**TABLE 1:** Emerging subphenotypes in adult TBI.

Study/platform	Cohort	Defining signature	Outcome or treatment effect	Reproducibility status
Multiplex screening of brain enriched and inflammatory proteins in serum/CSF; FlexMap 3D Luminex platform (Lindblad et al., 2021)	90 severe TBI patients and 15 control subjects	Suggested complement activation and inflammatory cytokine signals	BBB disruption associated with poor GOS scores; no treatment-effect link	Internal validation cohort for pathway and outcome-prediction findings
Immune-response endotypes; 30-analyte multiplex immunoassay (Samanta et al., 2024)	CENTER-TBI (discovery); TRACK-TBI Pilot (validation)	Two clusters: early-inflammatory vs. pauci-inflammatory; early-inflammatory ↑ IL-6, IL-15, MCP-1; parsimonious IL-15 + MCP-1 signature	Early-inflammatory cluster (older patients) associated with unfavourable GOS-E at 6 months; proposed for immunomodulatory enrichment	Clusters replicated across discovery + validation; parsimonious signature identified phenotype in both
Multi-omic injury-response endotypes; SomaScan proteomics, mass-spectrometry metabolomics/lipidomics, and endothelial markers (Wu et al., 2021)	Severely injured trauma incl. TBI (PAMPer-linked)	“Systemic storm” vs. “massive consumption” patterns; two integrated endotypes	Endotype-2 TBI benefits from prehospital thawed plasma (30-day mortality ∼30% vs. 75%); UCH-L1 proxy	Endotypes internally validated; treatment interaction single-cohort, not yet externally replicated
Plasma proteomic inflammatory responses; Alamar NULISA Inflammation panel (Li et al., 2026)	BIO-AX-TBI study participants (n = 37 acute moderate–severe TBI (Mayo Criteria), n = 22 acute non-TBI trauma (NTT), n = 28 non-injured controls (CON))	TBI-specific derangement in NF-κB, IL-1 and calcium regulation pathways	VSNL1, IL1RN/IL-1Ra, GFAP, and IKBKG identified as TBI-specific proteins; IL-33 (a broader, non-TBI-specific injury response) associated with 6-month functional outcome; no treatment-effect link	Exploratory, small cohort; not externally validated
High-dimensional plasma proteomic subtypes (BIO-AX-TBI); Olink Target 96 Inflammation + Alamar NULISA CNS Diseases panels (Li et al., 2025)	38 moderate–severe TBI (88 total w/controls); single timepoint ≤10 days	Neurodegenerative, inflammatory, and cellular-process proteins; three TBI-predominant subgroups (of five)	Subgroups map to injury pattern but not age or 6-month GOS-E; no treatment-effect link	Exploratory; small single cohort with cross validation through two platforms; subgroups not outcome-linked
Temporal proteomic trajectories of TBI CSF samples; mass spectrometry (Shultz et al., 2022)	91 CSF samples collected from 16 patients with severe TBI on day 0, 1, 2, 4, 7 and/or 10 post-injury; CSF from 11 non-injured controls	Persistent neutrophil and myeloid activation	7 proteins (GM2A, Calsyntenin 1, FAT2, GANAB, Lumican, NPTX1, SFRP2) were positively associated with an unfavourable outcome (GOS-E score) at 6 months post-injury; no treatment-effect link	Single-cohort, exploratory; not externally validated
Plasma proteomic trauma subtypes (torso vs. brain injury); Olink Explore 3072 (Tachino et al., 2024)	59 blunt-trauma patients, single-centre	Five endotypes (α-ε) by injury site/severity; coagulation and nerve-regeneration vs. systemic-adaptation proteins	Endotypes reflect injury location, severity, and prognosis	Single-cohort, exploratory; not externally validated
Data-driven clinical endotypes; unsupervised probabilistic clustering of clinical, physiological, laboratory, and imaging data (Åkerlund et al., 2022)	1728 ICU patients, CENTER-TBI	Six subphenotypes defined largely by systemic metabolic derangement (lactate, glucose, base excess)	Improved IMPACT prognostic model for outcome and mortality; no treatment-effect link	Internally stable (large single cohort); not externally validated
ICU disease trajectories; longitudinal clustering of daily clinical and physiological data (Åkerlund et al., 2024; Åkerlund et al., 2022)	CENTER-TBI ICU cohort	Six longitudinal ICU trajectory clusters (α–ζ), reflecting transitions in metabolic and physiological state	Distinct trajectories over ICU stay; subgroup membership depends on sampling time	Single consortium; reinforces temporal instability of subgroups

Each of these limitations has a practical remedy, although none is quick. Small single-cohort studies call for large multicentre observational cohorts, such as CENTER-TBI and TRACK-TBI, designed from the outset for external validation. Variation in sampling calls for shared protocols for sample type, processing, storage, and clock time, with fixed sampling windows. Variation across platforms calls for bridging studies and for reporting at the pathway level, which is less tied to any one assay. Data sharing calls for common data elements and a shared repository. Although FITBIR no longer accepts new submissions, its structure of common data elements linked to clinical data is a useful template for future biomarker studies (Thompson et al., 2015). Finally, links between subgroups and outcome, and between subgroups and treatment response, can only be established by prospective validation, ideally embedded within trials. Even with all of these in place, enrichment will not remove all variability. TBI outcome is shaped by a very large number of measured and unmeasured factors, from injury pattern and secondary insults to rehabilitation and social support, and randomization and stratification may still leave treatment groups imperfectly balanced, particularly in smaller trials. Residual confounding and unexplained variance may therefore continue to hide a real treatment effect, and enrichment should be seen as improving the odds of detecting one rather than guaranteeing it (Imberti, 2025).

### Neuroinflammation through the lens of high-dimensional omics

Neuroinflammation is a primary driver of secondary injury in TBI, perpetuated by activated glia, peripheral immune-cell recruitment, and inflammatory mediator release (Xiong et al., 2018). Given that anti-inflammatory agents have proven largely ineffective in clinical trials, the dual, context-dependent nature of the neuroinflammatory response demands deeper investigation (Russo and McGavern, 2016; CRASH Trial Collaborators, 2004). By resolving pathways rather than isolated proteins, high-dimensional omics have characterized dysregulated neuroinflammatory programs in accessible biofluids and linked them to patient phenotypes (Abu Hamdeh et al., 2021). We group the evidence below into six recurring pathway modules, each covered in one of the subsections that follow and summarized in Figure 2. A module here is a set of co-regulated proteins and pathways that tend to change together across studies. Certain pathobiological themes occur across platforms, including innate immune and complement activation, BBB breakdown, dysregulated neutrophil and myeloid effector programs, and a convergence with structural neuronal injury (Figure 2).

**FIGURE 2:**
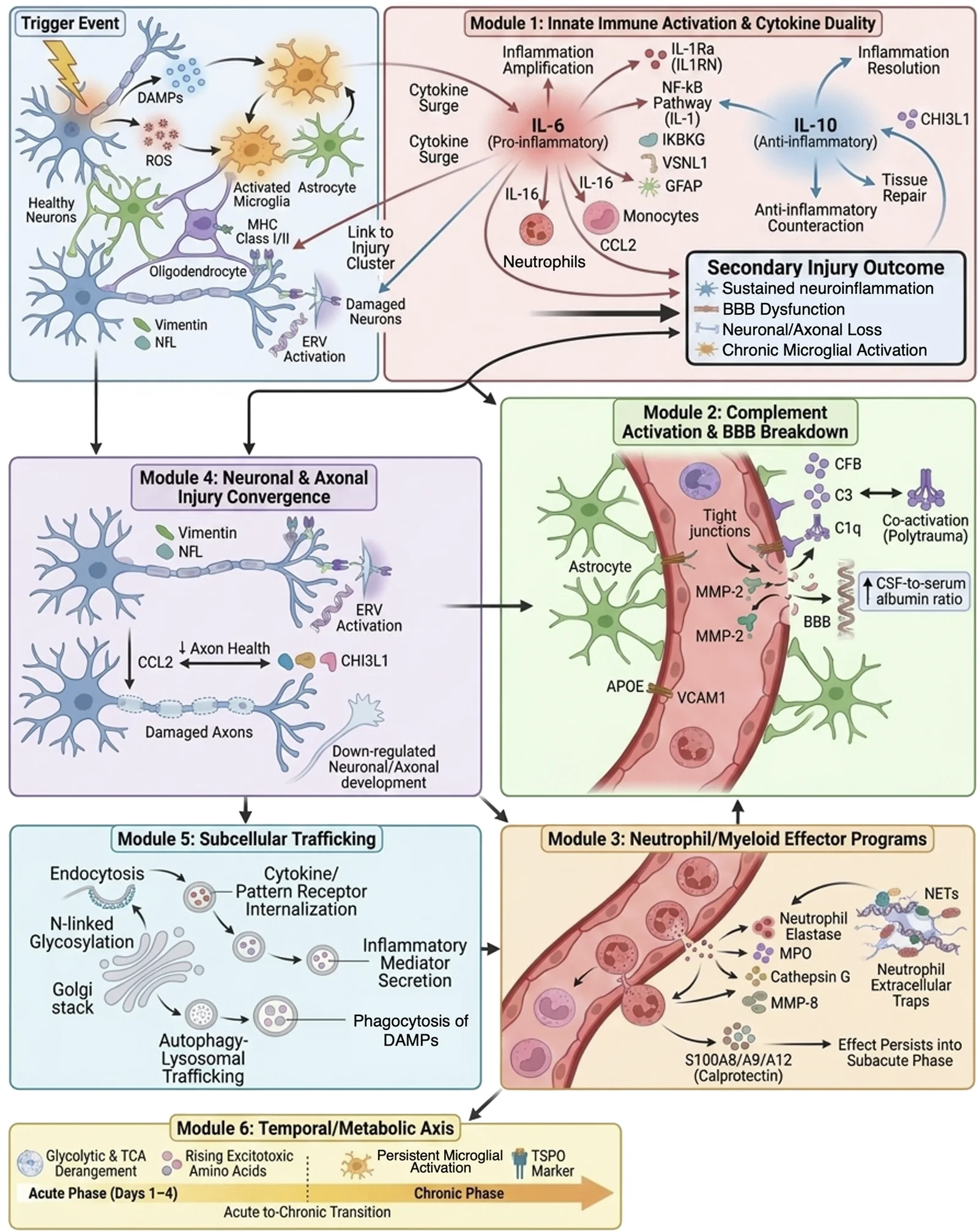
Convergent Neuroinflammatory Pathways In TBI Resolved By High-dimensional Omics. Mechanical injury releases damage-associated molecular patterns (DAMPs) and reactive oxygen species that activate microglia and astrocytes, initiating a coordinated cytokine response that is organized into six recurring pathway modules identified in this review. Module 1: innate immune activation and cytokine duality, pairs pro-inflammatory (IL-6) and anti-inflammatory (IL-10) hubs with IL-1/NF-κB signaling and IL-16/CCL2-driven immune-cell recruitment. Module 2: complement activation and blood-brain barrier (BBB) breakdown, links the complement cascade (CFB, C3, C1q) to MMP-mediated tight-junction loss. Module 3: neutrophil and myeloid effector programs, shows neutrophil degranulation (elastase, MPO, cathepsin G, MMP-8, S100A8/A9/A12) and NETs persisting into the subacute phase. Module 4: neuronal and axonal injury convergence, links a neuronal and glial injury signature (vimentin, NfL, CHI3L1) and CCL2-associated white-matter damage to an early oligodendroglial interferon response with ERV activation. Module 5: subcellular trafficking, implicates clathrin-mediated endocytosis, N-linked glycosylation, and endolysosomal machinery in cytokine handling and mediator secretion. Module 6: the temporal and metabolic axis, tracks an acute glycolytic-to-excitotoxic shift (days 1–4) that transitions to chronic microglial activation. All modules converge on sustained neuroinflammation, BBB dysfunction, neuronal and axonal loss, and chronic microglial activation. (DAMPs, damage-associated molecular patterns; NETs, neutrophil extracellular traps; ERV, endogenous retrovirus).

### Innate immune activation and cytokine duality

Following TBI, neuroinflammation is simultaneously injurious and reparative, reflecting the diverse functions of individual cytokines and their distinct temporal trajectories. Oxidative stress and damage-associated molecular patterns (DAMPs) released from injured cells typically trigger acute inflammation, glial activation and a cytokine surge (Zhu et al., 2026). High-dimensional proteomics resolves this at the pathway level. Exploratory PEA analyses in adults with severe TBI identify upregulation of Immune System, Innate Immune System, Cytokine Signaling, and Signaling by Interleukin Reactome pathways, with IL-6 and IL-10 emerging as two densely connected hub proteins in a network analysis (Van Nynatten et al., 2026d). The paired emergence of IL-6 (pro-inflammatory) and IL-10 (anti-inflammatory) hubs demonstrates the coexisting pro- and anti-inflammatory response that has confounded TBI clinical trials (Csuka et al., 1999; Ooi et al., 2022; Nwachuku et al., 2016).

Additional affinity-proteomic panels illustrate similar findings. In adults with moderate-to-severe TBI from the BIO-AX-TBI cohort (a prospective multicentre study that pairs blood biomarkers with diffusion MRI) the Alamar NULISA Inflammation panel isolated four proteins with TBI-specific elevation (visinin-like protein 1 (VSNL1), IL-1 receptor antagonist (IL1RN/IL-1Ra), GFAP, and the NF-κB regulator IKBKG), pointing to IL-1 and NF-κB signaling, with IL-1Ra correlating with white-matter injury (Li et al., 2026). Pairing the NULISA CNS Diseases panel with the Olink Target 96 Inflammation panel in the same cohort extended this to sixteen TBI-specific proteins, prominently the chemoattractants IL-16 and CCL2, which recruit peripheral immune cells and amplify neuroinflammation (Li et al., 2025). A concurrent anti-inflammatory response was also apparent, but as part of a broader injury reaction rather than a TBI-specific one. The anti-inflammatory mediators IL-10 and chitinase-3-like protein 1 (CHI3L1) rose after injury in both TBI and non-TBI trauma (Li et al., 2025). The parallel emergence of pro-inflammatory recruitment (IL-16, CCL2) and this anti-inflammatory counter-response again marks concurrent pro- and anti-inflammatory signaling as a core, reproducible feature of the TBI response.

### Complement activation and blood-brain barrier breakdown

Complement activation is the most reproducible innate-immune signal in adult human TBI proteomics, and is mechanistically linked to BBB disruption (Kossmann et al., 1997). In 90 adults with severe TBI, a suspension bead antibody array (FlexMap 3D Luminex) profiled 177 proteins in matched CSF and serum to investigate which brain-enriched and inflammatory proteins track BBB injury and 6-to-12-month outcomes (Lindblad et al., 2021). Elevated serum complement factor B and CSF complement components predicted worse functional outcome (unfavourable Glasgow Outcome Scale at 6–12 months), and complement proteins were the strongest correlates of the CSF-to-serum albumin quotient (a direct index of BBB integrity) with the lectin complement pathway enriched in CSF (Lindblad et al., 2021). The same samples showed increased IL-1α, IL-1β, and IL-6 alongside elevated matrix metalloproteinase-9 (MMP-9), which degrades tight-junction and matrix proteins, linking cytokine signaling to barrier breakdown (Lindblad et al., 2021). Mass-spectrometry CSF proteomics from 21 adults with acute TBI, compared with patients with infectious and non-infectious CNS conditions, investigated whether the CSF proteome separates these inflammatory states and corroborated a similar signature, with complement factor B (CFB), C3, and apolipoprotein E (APOE) distinguishing TBI from both control groups (Dyhrfort et al., 2024). In 232 patients meeting trauma-team activation, discovery plasma proteomics and metabolomics separated TBI from non-TBI trauma, with the proteins elevated in the severely injured polytrauma and polytrauma-TBI groups enriching for coagulation and complement-cascade activation and the analysis surfacing plasma markers of BBB disruption (APOE, vascular cell adhesion molecule-1 (VCAM1), MMP-2) (Cralley et al., 2023). Together, these findings position the complement-BBB axis as a convergent, outcome-associated node accessible in both CSF and blood.

### Neutrophil and myeloid effector programs

Unbiased proteomics also reveals a predominant neutrophil and myeloid effector response. In adults with severe TBI whose CSF was sampled serially from day 0 to day 10, high-resolution mass spectrometry suggested neutrophil recruitment, activation, and degranulation alongside broader myeloid activation, with neutrophil degranulation ranking among the top enriched pathways in both Gene Ontology and Reactome analyses (Shultz et al., 2022). The most highly upregulated proteins at each time point (neutrophil elastase, myeloperoxidase, cathepsin G, matrix metalloproteinase-8, and S100 calcium-binding proteins A8, A9, and A12) rose 5- to 10-log_2_fold over non-injured controls, and several (S100A9, S100A12, and cathepsin G) persisted into the subacute phase, pointing to a durable window for neutrophil-targeted, anti-inflammatory intervention (Shultz et al., 2022). Plasma proximity extension analysis recovers the same program systemically, identifying neutrophil degranulation as a significantly enriched pathway (Van Nynatten et al., 2026d). This concordance across intrathecal mass spectrometry and systemic affinity proteomics, in independent cohorts, marks granulocytic effector activity as a reproducible, compartment-spanning feature of acute TBI neuroinflammation (Shi et al., 2025).

### Convergence with neuronal and axonal injury

Neuroinflammation does not occur in isolation but is structurally coupled to neuronal and axonal injury within the underlying molecular network. In CSF samples from 16 adults with severe TBI, increasing neutrophil and myeloid gene programs were accompanied by downregulation of neuronal and axonal development pathways between days 1 and 10, underscoring the cost of inflammation to neural repair (Shultz et al., 2022). Exploratory network analysis of the adult plasma proteome has also supported this coupling of neuroinflammation with neuronal and axonal injury. An identified neuronal-injury cluster (vimentin, neurofilament light chain, and CHI3L1) interconnected with a cytokine-inflammation cluster through the IL-6 and IL-10 hubs (Van Nynatten et al., 2026d). Consistent with a direct inflammatory contribution to white-matter damage, acute plasma CCL2 in the BIO-AX-TBI cohort correlated inversely with corpus-callosum fractional anisotropy, linking a peripheral-recruitment chemokine to diffusion-MRI evidence of axonal disruption (Li et al., 2025). Single-cell transcriptomics of surgically evacuated tissue from patients with severe TBI adds additional cellular resolution. Transcriptomic analysis identified an early innate-immune response (an interferon signature with MHC class I and II induction) in oligodendrocyte precursors and mature oligodendroglia, together with activation of endogenous retroviruses. These findings implicate oligodendroglia, a non-classical immune cell type, in initiating neuroinflammation (Garza et al., 2023). Together, these convergent findings emphasize neuroinflammation as a driver of neuronal and axonal injury rather than a parallel process, making inflammation control and neuroprotection a single therapeutic objective.

### Subcellular trafficking

Pathway-level proteomics has also implicated subcellular membrane-trafficking machinery, a process not classically tied to neuroinflammation. In plasma from an exploratory cohort of adults with severe TBI, clathrin-mediated endocytosis, Golgi transport, and asparagine-linked glycosylation were among the most enriched pathways (Van Nynatten et al., 2026d). Moreover, these pathways tracked with markers of clinical severity, including ICU and hospital length of stay, hydrocephalus, and neurosurgical intervention, prompting the proposal of trafficking dysregulation as a candidate molecular endotype (Van Nynatten et al., 2026d). Indeed, vesicular transport is mechanistically inseparable from inflammation. Clathrin-mediated endocytosis regulates the internalization and signaling of cytokine and pattern-recognition receptors; Golgi trafficking and N-linked glycosylation enable the maturation and secretion of inflammatory mediators; and endolysosomal trafficking supports DAMP processing and phagocytic clearance by activated glia (Van Nynatten et al., 2026d; Husebye et al., 2006). Notably, membrane-trafficking dysregulation has independent support in TBI, as impaired autophagy–lysosomal clearance and altered serum and CSF N-glycosylation have both been reported after injury, although the specific clathrin- and Golgi-associated plasma signature remains to be replicated (Sarkar et al., 2014; Mondello et al., 2022).

### Temporal dynamics and the acute-to-chronic gap

Across platforms (Luminex bead arrays, affinity proteomics (Olink PEA, Alamar NULISA), and mass spectrometry) and fluids (CSF, blood), complement activation and innate-immune signaling are the most reproducible human proteomic signals in TBI, intersecting with BBB breakdown, astrocytic and axonal injury markers, and oxidative stress. However, temporal characterization remains limited. Most high-dimensional omics studies have focused on the acute and subacute phases of TBI, leaving the chronic molecular response poorly defined. Although translocator protein PET demonstrates persistent microglial activation for up to 17 years after injury (Ramlackhansingh et al., 2011), chronic TBI has largely been studied using imaging and targeted biomarker assays rather than unbiased multi-omic approaches. Emerging serum metabolomic studies in severe TBI reveal a temporal shift from acute glycolytic and TCA-cycle disturbances toward excitotoxic amino-acid signatures between days 1 and 4, highlighting the value of moving beyond proteomics toward integrated multi-omic reconstruction of disease trajectories (Banoei et al., 2025).

Which markers define the move from the acute to the chronic phase is not yet known. Current data point to candidates rather than a defined boundary, including the shift from a neutrophil-driven to a reparative CSF program over the first 10 days (Shultz et al., 2022), the day 1–4 metabolic shift described above (Banoei et al., 2025), and persistent microglial activation on PET (Ramlackhansingh et al., 2011). Defining this transition will require serial sampling that continues beyond the ICU stay. A related opportunity is to estimate the cellular source of bulk signals. Plasma proteomic data can be partly deconvolved using cell-type-enriched protein signatures drawn from single-cell atlases (Karlsson et al., 2021), and paired single-cell profiling of circulating immune cells could anchor these estimates and show how specific cell populations respond to therapy. Because plasma proteins can come from many tissues, the cell of origin will often remain uncertain, and these estimates should be treated as hypothesis-generating.

## From biology to better trials: pathway-based predictive enrichment

Failures in patient selection, inconsistent biological sampling, therapeutic timing, and mechanistic targeting all point to the same underlying challenge: insufficient biological enrichment. Two forms must be distinguished. Prognostic enrichment selects patients at higher risk of a poor outcome, concentrating events and improving statistical efficiency, but it does not identify who will respond to a given therapy (Stanski and Wong, 2020). Predictive enrichment, the more powerful and more demanding design, selects patients whose biology makes them more likely to benefit from a specific intervention (Stanski and Wong, 2020; Van Nynatten et al., 2026a). The TBI trial record is, in large part, a record of absent predictive enrichment. Agents were tested in all-comers defined by clinical severity, so that any effect confined to a biological subgroup was averaged into invisibility.

The hypothesis that predictive enrichment can rescue signal from a heterogeneous critical-illness syndrome is supported by evidence from sepsis and ARDS. Latent-class analysis of clinical and biological data repeatedly identifies two subphenotypes in ARDS (hyperinflammatory and hypoinflammatory) that differ in mortality and, crucially, in their response to randomized interventions. In retrospective analyses of completed trials, the subphenotypes responded differently to positive end-expiratory pressure and to fluid-management strategy, and in a secondary analysis of the HARP-2 trial the hyperinflammatory subphenotype derived a survival benefit from simvastatin that was absent in the cohort as a whole (Calfee et al., 2018; Calfee et al., 2014). Analogous treatment-responsive phenotypes have been described in sepsis (Seymour et al., 2019; Antcliffe et al., 2019). The lesson for TBI is twofold. Heterogeneity of treatment effect is the rule rather than the exception, and the same trials that read as null overall can contain real, recoverable benefit once the population is partitioned by biology. The immune-endotype and injury-endotype findings reviewed above (Samanta et al., 2024; Wu et al., 2021) indicate that TBI is amenable to the same approach.

Predictive enrichment selects patients most likely to respond to a therapy based on a measurable feature linked to its mechanism. In its simplest form, that feature is a single biomarker, where one analyte above a threshold marks a patient as eligible. Pathway-based enrichment works differently. Instead of asking whether one protein is above a threshold, it decides eligibility from the activity of a whole biological process (a group of molecules that act together, such as innate-immune and complement activation, or endothelial and coagulation signaling). This can be read out in two ways. One is to combine many members of the pathway into a single module-level score. The other, more practical at the bedside, is to measure a small parsimonious set of markers that best identify whether a patient belongs to the dysregulated pathway. A parsimonious panel comprises the fewest markers, typically two to five, needed to assign patients to pathway-defined groups with acceptable accuracy, as illustrated by the IL-15 and MCP-1 signature described above (Samanta et al., 2024). Either way, the feature selecting the patient is the state of the mechanism, not the level of any single marker (Van Nynatten et al., 2026b; Van Nynatten et al., 2026c; Van Nynatten et al., 2026a). Defined this way, pathway-based enrichment improves on single-marker enrichment on precisely the axes that have caused trouble (Van Nynatten et al., 2026a; Van Nynatten et al., 2026b). Because it is anchored to mechanism rather than correlation, this approach is less vulnerable to compartment effects and ceiling artifacts that distort individual analytes. By aggregating co-regulated biology, it is more stable across cohorts and platforms, and because the selection signature and therapeutic target can reside in the same pathway, patient stratification and mechanism of action are coupled by design rather than inferred after the fact. Operationally, each failure mode maps to a specific solution: heterogeneity requires molecular rather than purely clinical criteria; compartment effects require compartment-aware or paired-sample biomarkers and direct CNS target-engagement measures; temporal dynamics require time-windowed enrollment and serial sampling; and nonspecific targeting requires pathway-matched treatment allocation. These strategies appear technically feasible but remain to be tested prospectively, and the main challenge is designing trials around biology rather than clinical labels.

Feasibility differs sharply between the two readouts. High-dimensional profiling with proximity extension or NULISA panels needs only a small plasma volume, but samples are usually run in batches at a core facility or commercial provider, with turnaround of days to weeks, per-sample costs from roughly a hundred to over a thousand dollars depending on panel size, and dedicated bioinformatics support for quality control and pathway analysis. This suits discovery, retrospective subgroup analysis, and biology embedded in trials, but not enrollment decisions that must be made within hours. A parsimonious panel can instead be run on standard immunoassay or point-of-care platforms like those already used for GFAP and UCH-L1, with results in under an hour and little specialized staff (Bazarian et al., 2018). The practical path is to discover and validate pathway signatures on high-dimensional platforms and then translate them into rapid parsimonious assays for prospective enrollment and, eventually, clinical care.

Biomarkers can also serve as trial endpoints, but in a limited role. In early-phase trials, pathway markers measured in blood, CSF, or microdialysate are well suited as pharmacodynamic or target-engagement endpoints, showing that a drug reached and changed its intended pathway, as in the anakinra trial (Helmy et al., 2014). Using them as surrogate endpoints for efficacy is a much higher bar. No TBI biomarker has yet been shown to track treatment-related change in functional outcome well enough to replace it, so pivotal trials will still need clinical outcomes such as the GOS-E, with biomarkers as supporting evidence.

## Repurposing: a faster route to testable drugs in traumatic brain injury

Drug repurposing is the use of an existing, already-approved drug for a new indication different from the one it was originally developed to treat. Omics and informatics now drive this process by linking molecular signatures or dysregulated pathways to compounds known to act on them (Figure 3). Better patient selection raises the probability that a trial detects a true effect, while drug repurposing lowers the cost and time of generating the candidates worth testing. *De novo* CNS drug development is slow and expensive, and is burdened by an additional hurdle absent from most therapeutic areas; the need to cross the BBB at a therapeutic concentration (Alavijeh et al., 2005). Decades of investment in novel neuroprotectants have yielded little, and the attrition is unlikely to be overcome by new molecules alone (Maas et al., 2017). Repurposing offers a complementary strategy. Rather than designing a new agent for a nominated target, identify existing, often already CNS-penetrant drugs whose mechanism matches a dysregulated pathway in a defined patient subgroup, and test them with a known pharmacology and safety profile already in hand (Pushpakom et al., 2019).

**FIGURE 3:**
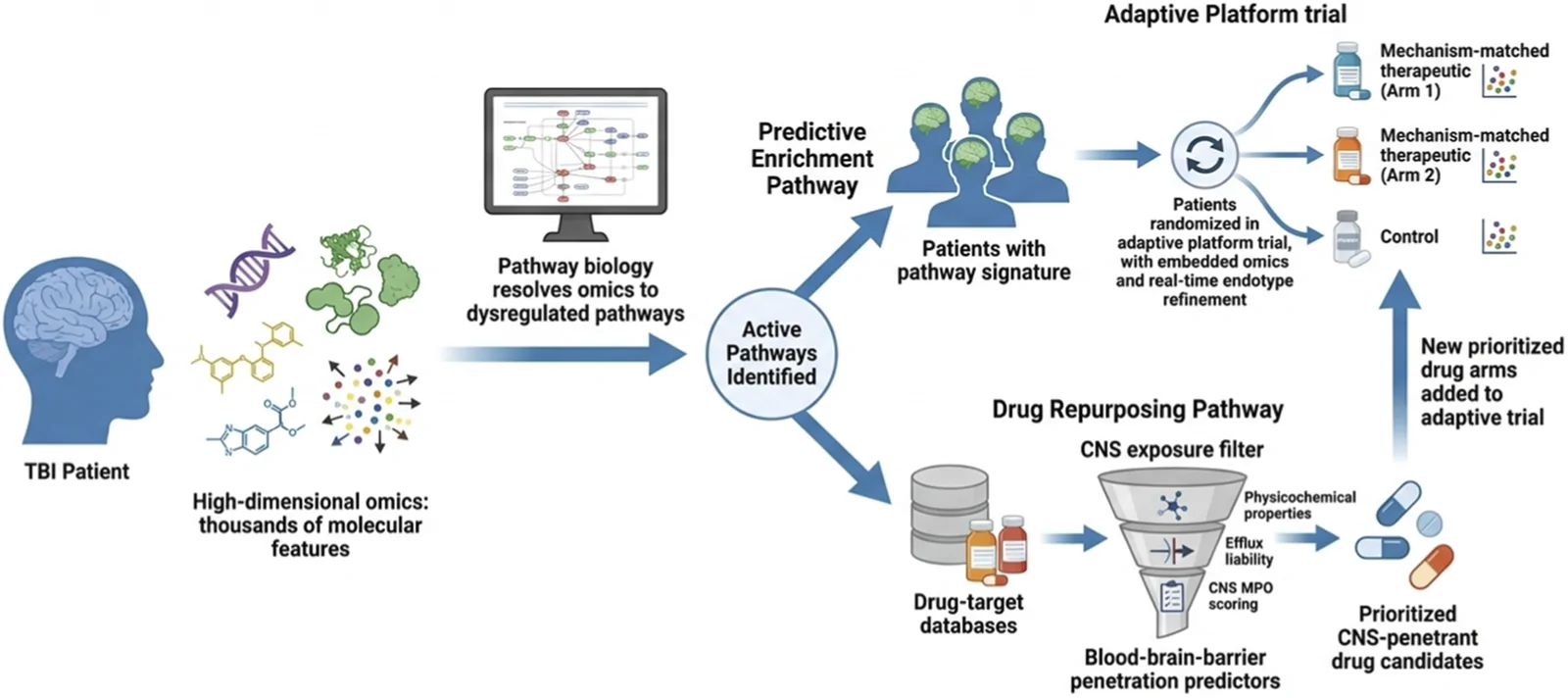
An Omics-guided, Pathway-based Pipeline Coupling Predictive-enrichment Trials With Drug Repurposing In Traumatic Brain Injury. High-dimensional omics profiles thousands of molecular features in each TBI patient, which pathway biology resolves into the dysregulated, active pathways driving injury. Each active pathway is then used in two coupled directions. For patient selection (predictive enrichment), patients carrying the pathway signature are enrolled into an adaptive platform trial and randomized between mechanism-matched therapeutic arms and control, with omics embedded in the trial so profiling continues and endotype definitions are refined in real time. For therapy selection (drug repurposing), the same pathway is screened against drug-target databases to identify existing, already-approved agents that modulate it, and this shortlist is passed through a CNS-exposure filter using blood-brain-barrier (BBB) penetration predictors (physicochemical properties, efflux-transporter liability, and *in silico* CNS multiparameter optimization [MPO] scoring) to prioritize candidates able to reach an intracranial target. These prioritized, CNS-penetrant drugs are added as new arms to the adaptive platform trial, closing a loop in which the same biology selects both the patients enrolled and the therapies they receive.

Pathway-level omics offers a practical way to match drugs to patients for this purpose (Van Nynatten et al., 2026b). A pathway found to be dysregulated in a molecularly defined subgroup can be connected to compounds known to modulate it, narrowing a vast drug library to a short list of mechanism-appropriate, repositionable candidates (Lamb et al., 2006; Subramanian et al., 2017). That shortlist can then be filtered by the constraint specific to neurotherapeutics (CNS exposure) using established predictors of blood-brain barrier penetrance (physicochemical properties, efflux-transporter liability, and *in silico* CNS multiparameter optimization scoring) to prioritize agents likely to reach an intracranial target, before experimental confirmation of target engagement (Wager et al., 2010). The sequence is therefore 1) omics nominates the active pathway, 2) the pathway nominates mechanism-matched drugs, and 3) BBB-penetrance modeling prioritizes those able to act within the CNS. Table 2 catalogues the major agents tested in adult TBI, the trial evidence for each, the failure mode that most plausibly explains a null result, and the corresponding enrichment or repurposing opportunity. Read through this lens, several historical “failures” are better understood as untested hypotheses (mechanisms evaluated in unselected populations) that may warrant re-evaluation in pathway-defined subgroups rather than permanent abandonment.

**TABLE 2:** Major pharmacologic agents investigated for treatment effect in TBI.

Agent	Target/mechanism	Key trial (phase)	Result	Likely failure mode	Enrichment or repurposing opportunity
Methylprednisolone	Broad glucocorticoid immunosuppression	CRASH (III) (CRASH Trial Collaborators, 2004)	Increased mortality	Undifferentiated targeting; wrong window	Replace broad suppression with pathway-specific anti-inflammatory in an inflammation-high endotype
Progesterone	Pleiotropic neurosteroid (anti-inflammatory, anti-edema)	PROTECT III; SYNAPSE (III) (Wright et al., 2014; Skolnick et al., 2014)	No benefit	Heterogeneity; undifferentiated targeting	Re-evaluate in a neuroinflammation/edema-defined subgroup
Erythropoietin	Anti-apoptotic/neuroprotective; haematopoietic	EPO-TBI (III) (Nichol et al., 2015)	No neurological benefit	Heterogeneity; wrong mechanism in all-comers	Enrich for relevant injury/anaemia biology; pathway-defined responders
Citicoline	Membrane phospholipid precursor	COBRIT (III) (Zafonte et al., 2012)	No benefit	Heterogeneity; weak target specificity	Metabolic/membrane-injury–defined subgroup
Dexanabinol	NMDA antagonist/antioxidant/anti-TNF	Maas et al. (III) (Maas et al., 2006)	No benefit	Wrong window; undifferentiated targeting	Excitotoxicity/inflammation-high, early-window enrichment
Hypothermia	Temperature management (prophylactic; or ICP-directed cooling)	POLAR (prophylactic); Eurotherm3235 (ICP-directed) (III) (Cooper et al., 2018; Andrews et al., 2015)	No benefit (POLAR); harm (Eurotherm3235)	Wrong window; one-size dosing	Metabolic-crisis–defined subgroup; tighter timing windows
Tranexamic acid	Antifibrinolytic (stabilizes clot; limits intracranial bleeding)	CRASH-3 (III) (Crash-3 Trial Collaborators, 2019)	No overall benefit; reduced head-injury death in a prespecified mild-to-moderate subgroup (GCS 9–15)	Heterogeneity of treatment effect; benefit averaged out in all-comers	Enrich for mild-to-moderate injury and early (<3 h) treatment; target bleeding/coagulopathy biology
Anakinra (IL-1ra)	Interleukin-1 receptor antagonism (repurposed)	Helmy et al. (II) (Helmy et al., 2014)	Safe; CNS target engagement shown	Not applicable: feasibility trial	Enrich for IL-1/inflammasome-high endotype; verify engagement by microdialysis
Amantadine	Dopaminergic/NMDA modulation	Giacino et al. (II/III) (Giacino et al., 2012)	Accelerated recovery	Not applicable: weakly positive signal for benefit	Enrich for a dopaminergic/arousal-deficit endotype; use omics to identify likely responders and extend beyond clinically defined disorders of consciousness

Omics-guided drug repurposing is not a source of new drug efficacy but a way around two bottlenecks that have kept neurotherapeutic development barren. The first is discovery. Building a CNS drug from scratch is slow and costly; it has to cross the blood-brain barrier, and in acute injury it must act within a treatment window of only hours. These constraints favor drugs that already exist, already carry a known safety profile, and ideally are already known to reach the brain. The second is selection. Almost all past trials tested a mechanism in clinically defined all-comers, whereas pathway-level omics matches a drug to the specific mechanism active in a molecularly defined subgroup, rather than to the syndrome as a whole (Pushpakom et al., 2019). Addressing both does not guarantee success, but it improves the odds of detecting a real effect, if one exists, and lowers the cost and time of moving a credible candidate into a properly enriched trial. That promise is real but conditional. The approach is only as reliable as the subgroup definitions feeding it, and in TBI those definitions are not yet stable across cohorts, panels, and timepoints. Its value therefore rises and falls with the maturity of the upstream endotyping, which is why the near-term priority is to make those pathway-derived subphenotypes robust before using them to allocate therapy.

Repeated TBI trial failures have weakened industry investment, while repurposed generic drugs offer limited commercial incentives. Anakinra illustrates the gap: despite being licensed and showing target engagement in the injured brain, it has not advanced to a larger TBI trial since its phase II study (Helmy et al., 2014). Biological activity alone may therefore be insufficient to secure development. Progress may depend on publicly funded trial networks, adaptive platforms, and support for repurposing from funders and regulators (Imberti, 2025).

## Synthesis and outlook

The shortcomings catalogued here share a single root and suggest a path forward. A condition that is heterogeneous, compartmentalized, temporally dynamic, and mechanistically diverse cannot be matched to therapy through single markers, single mechanisms, and single timepoints. It requires a framework that specifies the right patient (by molecular endotype), the right sample (interpreted in a compartment-aware way), the right time (by trajectory rather than a single snapshot), and the right mechanism (pathway-matched, and where possible drug-repurposed). Such a framework must be tested under predictive enrichment rather than in clinically defined all-comers. Each element of this framework now has at least proof-of-concept support in human data. This includes validated immune endotypes, a demonstrated treatment-effect interaction in TBI subgroups, an increasingly convergent and reproducible pathway biology, and a maturing toolkit of brain-enriched biomarkers and intracranial readouts.

The remaining challenge is the prospective integration of these elements into trial design. Most endotype-to-treatment links remain to be confirmed. Multi-compartment, serially sampled datasets are still scarce, pathway signatures are rarely validated across independent cohorts, and CNS target engagement is almost never measured directly. The near-term priorities follow from these gaps. First, assemble paired blood-CSF, longitudinally sampled, high-plex cohorts, powered for cross-cohort validation. Critically, these cohorts must be built on high-sensitivity, CNS-directed assays that can detect low-abundance, brain-enriched proteins in blood; only that sensitivity separates genuine neural signal from the systemic response, and without it no blood-based signature can be trusted to reflect intracranial biology. Second, report omics at the level of pathways and modules, with explicit replication, rather than as ranked lists of individual analytes. Third, design the next-generation of therapeutic trials around pathway-defined, enrichment-selected populations, testing mechanism-matched and often repurposed agents, with embedded omic profiling both to verify CNS target engagement and to detect heterogeneity of treatment effect across molecular subgroups. The drugs that failed the syndrome of “TBI” may yet succeed in the right biology. The task is to characterize patients with a resolution that allows their biology to guide intervention.

## Glossary

APOE: apolipoprotein E
ARDS: acute respiratory distress syndrome
BBB: blood-brain barrier
CCL2: C-C motif chemokine ligand 2
CFB: complement factor B
C1q: complement component 1q
C3: complement component 3
CHI3L1: chitinase-3-like protein 1
CNS: central nervous system
CSF: cerebrospinal fluid
CT: computed tomography
DAMPs: damage-associated molecular patterns
DTI: diffusion tensor imaging
ELISA: enzyme-linked immunosorbent assay
ERV: endogenous retrovirus
FITBIR: Federal Interagency Traumatic Brain Injury Research
FA: fractional anisotropy
GCS: Glasgow Coma Scale
GFAP: glial fibrillary acidic protein
GOS: Glasgow Outcome Scale
GOSE: Glasgow Outcome Scale–Extended
ICP: intracranial pressure
ICU: intensive care unit
IKBKG: inhibitor of nuclear factor kappa-B kinase regulatory subunit gamma
IL: interleukin
IL-1Ra (IL1RN): interleukin-1 receptor antagonist
MHC: major histocompatibility complex
MMP: matrix metalloproteinase
MPO: myeloperoxidase
MRI: magnetic resonance imaging
MS: mass spectrometry
NETs: neutrophil extracellular traps
NF-κB: nuclear factor kappa B
NfL: neurofilament light chain
NULISA: nucleic acid-linked immunosandwich assay
PEA: proximity extension assay
PET: positron emission tomography
ROS: reactive oxygen species
S100A8/A9/A12: S100 calcium-binding proteins A8 A9 and A12
S100B: S100 calcium-binding protein B
TBI: traumatic brain injury
TCA: tricarboxylic acid
TSPO: translocator protein
UCH-L1: ubiquitin C-terminal hydrolase L1
VCAM1: vascular cell adhesion molecule 1
VSNL1: visinin-like protein 1
